# Supplementary figures and images for: Molecular Characterization of the Acyl-CoA-Binding Protein Genes Reveals Their Significant Roles in Oil Accumulation and Abiotic Stress Response in Cotton
Source: Genes (Basel). 2023 Apr 1;14(4):859. doi: 10.3390/genes14040859 (PMC10137972; doi:10.3390/genes14040859)

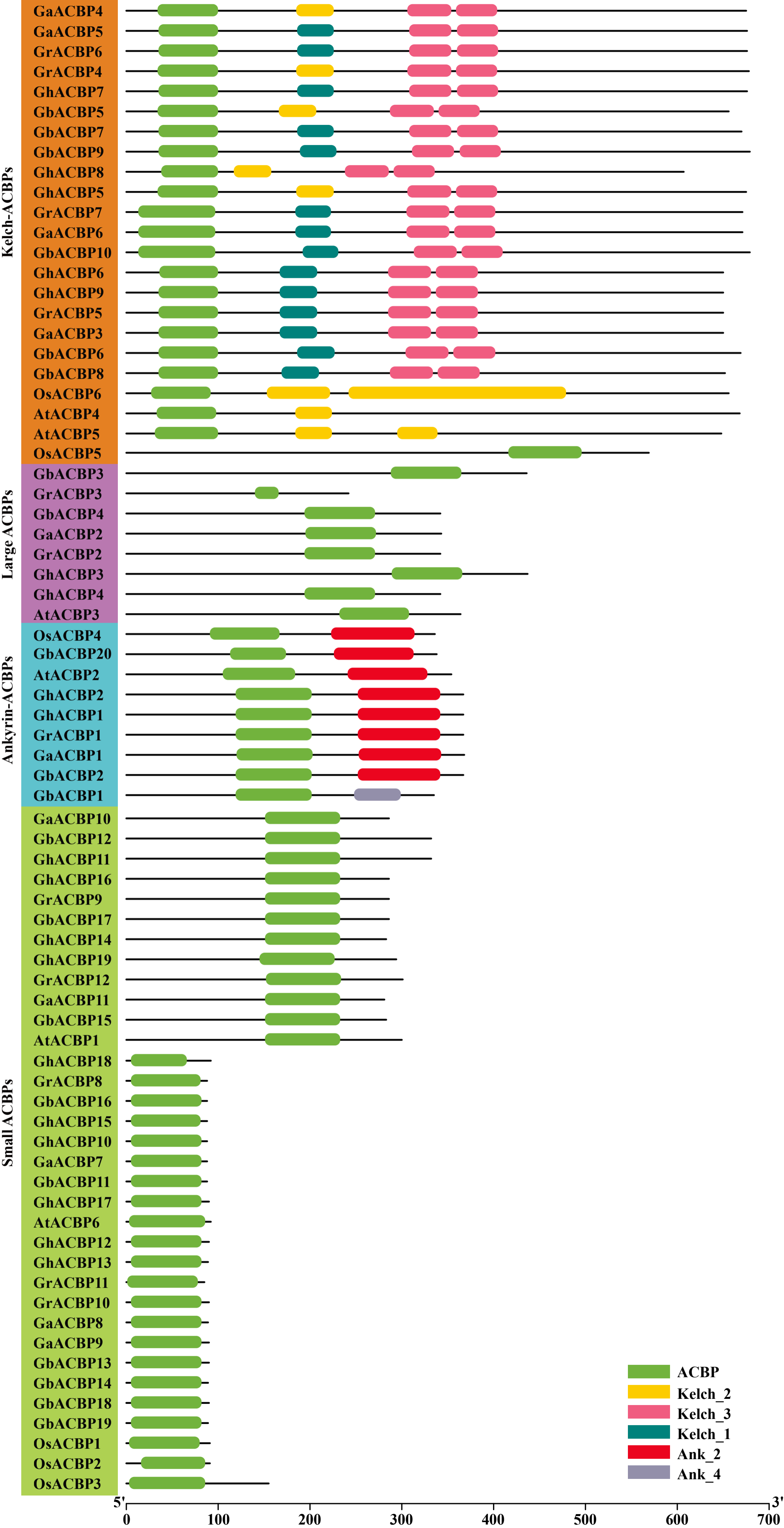

Supplement: Supplementary file 1 [file genes-14-00859-s001.zip › Figure S1. The conserved domains identified in cotton ACBP proteins.pdf]

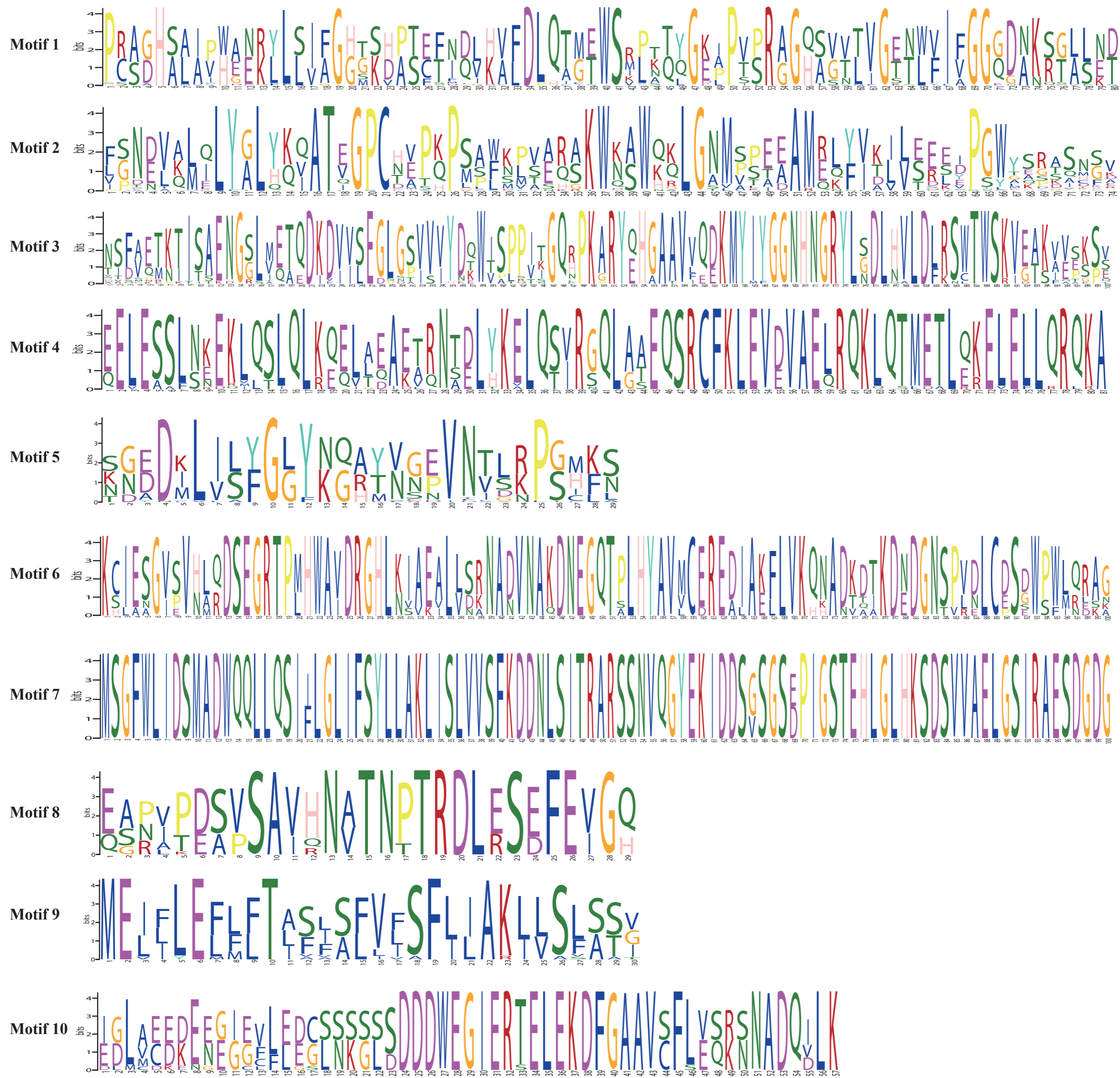

Supplement: Supplementary file 1 [file genes-14-00859-s001.zip › Figure S2. The consensus sequences of the ten conserved motifs predicted in GhACBP proteins.pdf]

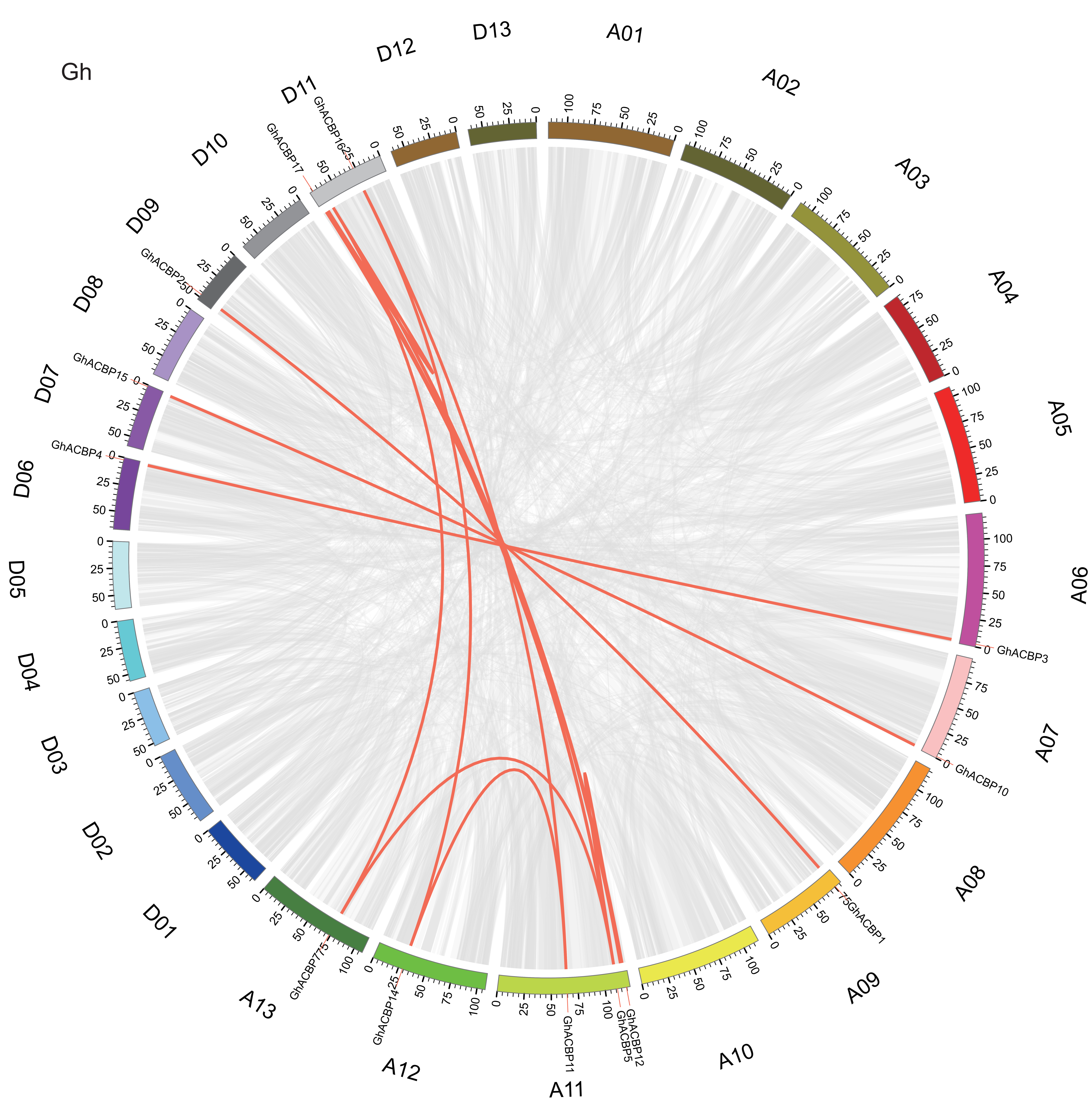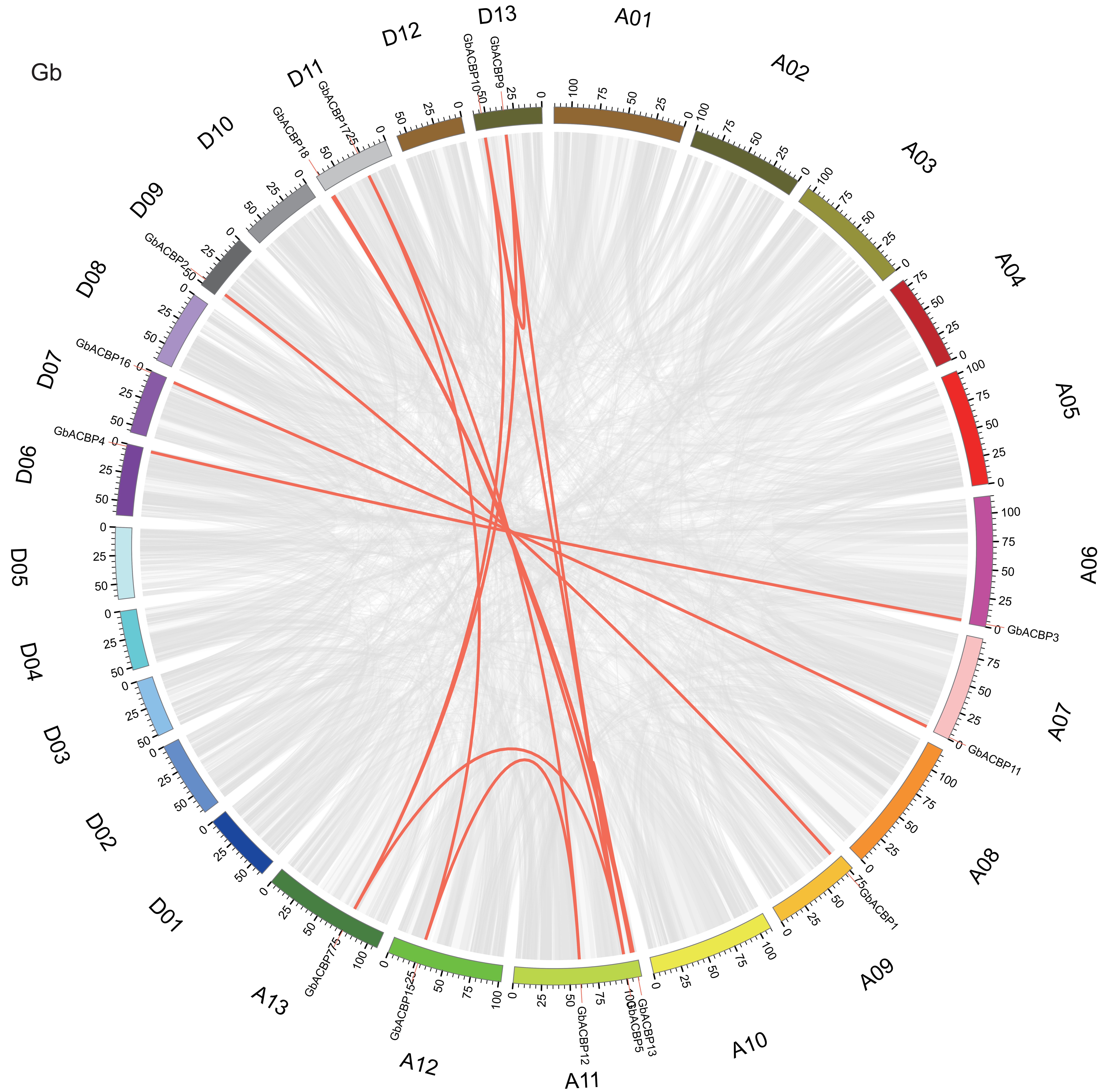

Supplement: Supplementary file 1 [file genes-14-00859-s001.zip › Figure S3. Circos diagram of the ACBP duplication pairs in Gossypium hirsutum and G. barbadense.pdf]
